# Has digital finance widened the income gap?

**DOI:** 10.1371/journal.pone.0263915

**Published:** 2022-02-14

**Authors:** Lianying Yao, Xiaoxiao Ma

**Affiliations:** 1 School of Economics, Zhejiang University of Technology, Hangzhou, China; 2 Global Institute for Zhejiang Merchants Development, Zhejiang University of Technology, Hangzhou, China; 3 School of Economics and Management, Si Chuan University, Chengdu, China; Politecnico di Torino, ITALY

## Abstract

Using the statistical data of 280 prefectural-level cities in China from 2011 to 2020, this paper empirically tests the relationship between digital finance and residents’ income in a linear and nonlinear model based on the G-J model theory, respectively. The study aims to discuss and analyze the impact of digital finance development on income distribution in the context of the current situation of digital finance development in China and further explore how to make digital finance better regulate the income distribution of residents. The innovation of this paper is to use two nonlinear methods to verify the Kuznets effect and threshold characteristics of digital financial development affecting the income distribution of residents based on linear analysis and explore the relationship between n digital economic development the current income gap more comprehensively. The study shows a Kuznets effect of digital finance development on the income distribution of Chinese residents. Thus, most regions in China have not yet crossed the inflection point of the bell-shaped curve, and the income gap within areas will continue to increase with the development of digital finance. By constructing a threshold model, it is found that the positive effect of digital finance on income disparity may initially increase with the increase of regional economic level. Still, when the regional economic development reaches a higher stage, the effect will tend to fall back. As a result, the negative impact of digital finance development on residents’ income distribution will be significantly reduced at that time.

## I. Introduction

As a product of combining digital technology with traditional finance, digital finance necessarily has financial attributes that no longer rely on the physical channels that traditional finance depends on, are more penetrating at the geographic level, and have lower cost advantages [1–3]. Based on theories of financial development and income distribution, we find that academics seek consensus and exploration and extension of the issue, but all acknowledge the critical role that finance plays in income distribution [4, 5]. In the era of the digital economy, digital finance has become an essential engine of financial development everywhere. Thus, it plays a vital role in both inter-regional development and intra-regional development. However, different scholars have different views on the impact of digital finance development on the current domestic income distribution [6–8]. Some scholars argue that the inclusive nature of digital finance significantly reduces the gap in income distribution, that access to payments, credit, and digital financial services in rural areas will contribute substantially to intra-regional inclusive growth, and that digital payment instruments can effectively converge the current urban-rural income gap in China [9, 10].

In contrast, digital finance has more than a simple linear impact on the income distribution of the population, but there are some nonlinear features such as threshold characteristics [11, 12]. Since digital technologies that drive inclusive financial development have thresholds such as infrastructure, practical application, and institutional environment, the existence of threshold effects allows digital economic growth to have different impacts on income distribution in other regions and financial exclusion [13–15]. The integration of inclusive finance and digital technologies is insufficient, and low-income groups still face multiple difficulties in accessing essential financial services. We found that low-income people tend to have less education and limited knowledge of finance and the Internet and that digital finance relies on a critical vehicle of electronic devices. A significant proportion of low-income groups have unequal access to digital financial applications due to their lack of access to appropriate electronic devices or inability to acquire proper Internet knowledge [16, 17]. A concept that must be mentioned here is the digital divide. The inequality in income and education levels of different population segments implies a gap in their access to and processing of information. The digital divide will exacerbate the information blockage of information-poor groups, limiting their opportunities and ways to achieve income generation and creating a new type of stratification. Thus, the essence of the digital divide is an economic divide [18–20].

Collating the current research results, it is easy to find that the existing literature systematically arguing the relationship between digital finance development and income distribution of residents is relatively limited, and there is still room for further exploration in terms of theoretical and empirical studies [6, 11, 21] Therefore, based on the existing literature, this paper mainly measures the Gini coefficient of income of each prefecture-level city in China and discusses and analyzes the impact of digital finance development on domestic income distribution in the context of the current situation of digital finance development in China, and further explores how to make digital finance better regulate the income distribution in China, to contribute to cracking the currently faced "uneven distribution The paper will discuss and analyze the impact of digital finance development on domestic income distribution, and further explore how digital finance can better regulate income distribution in China, and contribute to solving the current problem of "uneven distribution".

Compared with existing studies, this paper may have the following innovation: most current articles on digital finance and income distribution discuss the linear relationship between the two. At the same time, some representative literature advocates another view that the relationship between digital finance development and income distribution may have nonlinear characteristics. However, considering the current situation of digital finance development and income distribution in China and the related mechanism of digital finance development on income distribution, it is reasonable to suspect that the two are linearly related and probably have a more complex inner connection. Therefore, based on the linear analysis, this paper adopts two nonlinear methods to verify the Kuznets effect and threshold characteristics of digital financial development affecting the income distribution of residents, to explore the relationship between digital economic growth and the current income gap more comprehensively, and to explain the recent phenomenon of "digital divide" in China.

In addition, the current literature on income disparity mainly focuses on urban and rural areas. At the same time, the Gini coefficient measures the overall regional income disparity, and there is almost no research on the relationship between digital finance and the general regional income distribution. In this paper, we use the Gini coefficient, the most common international indicator, to measure the imbalance of regional income distribution [22, 23]. We use the current Gini coefficients of urban, rural, and all residents in 280 prefecture-level cities to explore the impact of the recent digital finance development on China’s income distribution and fill the gap in the current research on the relationship between digital finance and overall income distribution in China.

## II. Literature review

### 1. Study on the relationship between digital finance and income distribution

(Ren et al., 2018) [24] used the Peking University Digital Inclusive Finance Index to explore the mechanism of the current digital inclusive policies implemented in China on the income distribution of urban and rural residents. The development and application of digital technology dramatically improve the current income of residents. Its effect on the income improvement of urban residents is more prominent and has a heterogeneous impact on the income improvement of different groups in urban and rural areas [25]. Found that the implementation and development of regional digital finance can significantly increase the income level of residents [26]. Used panel data at the provincial level in China from 2011–2015 to confirm through an econometric model that the current digital inclusive finance in China has a positive impact on narrowing the income gap between urban and rural areas within regions [27]. Compiled data from 2004–2014 in China and applied the VECM model to find that digital financial development can effectively promote the development of inclusive finance within regions in China. The increasing level of inclusive finance development has contributed significantly to the equalization of income distribution in China [28, 29]. Argue that the digitalization of traditional industries only benefits the wealthy class in that industry, which will lead to more significant class differentiation. [30], who argue that the "digital divide" between countries around the world, between regions in China, and within areas should be guarded against as a challenge for governments at all levels as digital technology continues to evolve. As we can see, many scholars have pointed out that the development of digital technology is likely to widen the gap of income distribution based on the previous one. However, other scholars, such as [31, 32], use data from less developed countries to demonstrate that mobile network coverage can help markets function better and improve the welfare of low-income people.

### 2. Studies related to digital finance and rural poverty reduction

[33] conducted an empirical study on the development of communication infrastructure and farmers’ income in China and found that the increase in the availability of basic communication equipment in China did not improve the current income level of farmers and that there was "digital exclusion" in rural areas [34]. Found that the effective transmission of information in the digital financial system significantly increased the selling price of fresh agricultural products. Therefore the effective provision of timely and accurate market information is important for improving farmers’ effectiveness in the current digital environment, which is the key to narrowing the digital divide [35]. Found that rural laborers who use digital networks are more likely to be employed off-farm than those who do not use digital networks. The use of the Internet greatly increases their probability of starting a business, which contributes to a considerable extent to the increase in farmers’ income and effectively alleviates the gap between the rich and the poor between urban and rural areas [36]. Focused on poverty concentration zones in western China and found that the development of digital finance in poor regions has a single threshold characteristic effect on the incidence of local poverty [37]. Examined the effect of digital finance policies in China at both county and inter-provincial levels and found that it played a vital role in reducing the urban-rural income gap at the early stage of digital finance development. Still, this effect was only concentrated in the contiguous special hardship zones. Due to the lack of financial knowledge of rural groups, the phenomenon of "elite capture" of agricultural loans is serious, and the poverty benefit of digital finance-related policies has not been significantly reflected.

Early on [38], argues that digital finance can create a "digital divide" among low-income populations. [38–41], using relevant data from developing countries in Asia and Africa, mobile network coverage effectively improved the operational efficiency of agricultural markets, increased the price of locally sold agricultural products, and significantly improved the welfare of both parties to the transaction. This significantly improved the welfare of both parties involved in the transaction, resulting in a significant increase in the intra-regional economy. [42], focusing on Internet development in rural areas, most rural residents suffer from a high level of digital financial exclusion, with self-exclusion being the main factor in digital exclusion. In addition, the article also finds that farmers who are excluded by traditional credit are also more vulnerable to Internet credit exclusion, and it can be argued that poor farmers benefit from digital financial development with little impact [43]. Argues that the increased level of financial inclusion brought about by digital finance helps to reduce poverty, thereby reducing the income distribution gap.

### 3. Path mechanisms of digital finance affecting income distribution

Employment dimension: During the development of digital finance, rural residents’ access to credit has been broadened, and using the Internet, rural residents can start their businesses at a lower cost than before. The development of digital finance has, to some extent, increased the viability of entrepreneurship among rural groups, significantly alleviating their poverty-causing nature in this dimension and increasing farmers’ income. Many scholars have focused on the profound impact of digital finance development on the employment of the population [44]. Focused on the relationship between female entrepreneurship and Internet use, which proved that the use of the Internet significantly contributed to the overall employment of the women group and that the use of the Internet could effectively help married, low-education, and agricultural women to obtain employment [45]. Used a multivariate Probit model to verify the impact of Internet use on individual employee behavior and employment quality, and found that Internet use significantly increased the likelihood of individuals engaging in standard employment and opportunity entrepreneurship, but had the potential to widen regional due to the low Internet use skills of the poor as well as disadvantaged groups [46] studied the impact of regional Internet availability on local productive life using national data and concluded that intra-regional Internet penetration is effective in increasing local employment levels and that this effect is more pronounced in less developed rural areas. [47], using county-level data from the United States, households’ access to the Internet significantly increased their probability of employment. This effect was also more pronounced in rural and remote areas. Other scholars have argued that the mass use of smart devices in the digital economy will impact the employment of low-skilled workers and may lead to the marginalization of low-skilled workers, resulting in a widening of the income distribution gap [48]. Demonstrated through an empirical study that the increase in the use of industrial machines between 1990 and 2007 was an important reason for the deterioration of the local labor job market in the U.S. The number of machine applications is closely related to the corresponding pay of workers. The increase in their number will lead to a decrease in the average salary of workers. In contrast [49], argue that emerging technologies such as artificial intelligence and machine learning do not significantly affect the current division of labor and labor market.

Credit level: the inclusive digital finance that has been landing in the process of digital finance development has dramatically alleviated the credit constraints of residents in rural areas and the financing constraints of small enterprises, which is conducive to widening the employment channels of low-income groups. Therefore, many scholars have explored the mechanism of the Internet’s effect on income distribution in terms of its impact on credit constraints [50], empirically verified the impact of digital finance on the credit constraint of farmers’ groups and demonstrated the positive driving impact of digital finance on the entrepreneurship of rural groups [51]. Confirmed that digital finance significantly alleviates the credit constraints faced by households in general and found that it has more significant consumption incentives for families in less developed regions through heterogeneity analysis [7]. Suggest that "crowdfunding" by digital finance can complement traditional entrepreneurial financing methods.

### 4. Literature review

The current literature consistently defines digital finance’s connotation and affirms its significance to the recent social development. Digital technology provides a practical solution for the current financial growth in inclusive development. The advantages it shows in supporting the development of inclusive finance significantly reduce the distance between various groups enjoying financial services. The vast majority of scholars affirm its inclusive benefits. There is a consensus that digital economic development can raise urban and rural residents per capita disposable income. Still, the level of income enhancement for different groups of people is not the same, which has led to the consideration of the impact on the income distribution gap. Currently, academics hold the following three views on the relationship between digital finance development and income distribution: The first type is that digital finance development will reduce the income gap. This view is usually based on the inclusive nature of digital finance, which is believed to have a more substantial effect on raising the income of rural people and low-income groups, thus significantly reducing the income gap between urban and rural residents. The second type of view is that the development of digital finance will increase the income gap. It is believed that digital finance is more beneficial to the affluent class, and the "digital divide" will appear between low-income and high-income groups due to the existence of digital technology, which requires our vigilance. The third type of view is that the relationship between the development of digital finance and the income gap shows a Kuznets effect. There are not many studies of this kind.

The critical impact of digital finance development on the current income distribution has become a consensus in the academic field, so this paper carries out a theoretical and empirical study on the real issue of digital finance and income distribution based on the previous studies. The depth of digital finance use and the regional Gini coefficient is used to measure the development of digital finance and the regional income distribution, respectively, to conduct a comprehensive analysis of the current situation of digital finance development and income inequality of residents, and to analyze the inner mechanism of the actual situation. Furthermore, based on the data of prefecture-level cities, we explore the nonlinear relationship between digital finance development and income inequality and introduce the threshold model to analyze the threshold effect of digital finance development in different regional economic development levels, refine the impact of its role in various economic levels, which is closer to the current national situation, and provide more accurate guidance for national policy formulation.

## III. Method

### 1. Variable selection

#### (1) Income disparity (GINI)

Gini coefficient is the current internationally recognized authoritative indicator that can measure the imbalance of income distribution in a country or region; this paper uses the statistical yearbook of each prefecture-level city to divide the income into five equal groups to measure the Gini coefficient, the larger the GINI value represents the greater the degree of income distribution imbalance within the region.

In this paper, the Gini coefficient is measured using the five equal groups of income in the statistical yearbooks of each prefecture-level city. In the statistical yearbook of each prefecture-level city in China, the relative income disparity between urban and rural areas is divided into five equally spaced groups, from lowest to highest: lowest, lower, middle, higher and highest, and the Gini coefficient of each prefecture-level city are derived from the following equation.


$$G=1-\frac{1}{PW}\sum_{i=1}^{n}\left(W_{i-1}+W_{i}\right)\times P_{i}$$

*P* denotes the overall population of the area; *W* denotes the population’s total income; *W*_*i*_ represents the cumulative income sum to group i.

In this paper, we use the above formula to calculate the Gini coefficients of urban and rural residents’ incomes for 280 prefectures and use the "group weighting method" to calculate the Gini coefficients of residents’ incomes for each prefecture-level city as a whole.


$$G=P_{c}^{2}\frac{u_{c}}{u}G_{c}+P_{r}^{2}\frac{u_{r}}{u}G_{r}+P_{c}P_{r}\frac{u_{c}-u_{r}}{u}$$

Where: *P*_*c*_ represents the proportion of the urban population to the total population; *u*_*c*_ represents the per capita income of urban residents; *G*_*c*_ represents the Gini coefficient of urban residents’ income of each prefecture-level city; *P*_*r*_ represents the proportion of the rural population to the total population; *u*_*r*_ represents the per capita income of rural residents; *G*_*r*_ represents the Gini coefficient of rural residents’ income for each prefecture-level city. *u* Represents the overall per capita income of each prefecture-level city.

Using the above equation, this paper calculates the Gini coefficient of all residents’ income for 280 prefecture-level cities from 2011 to 2020.

#### (2) Level of digital finance development (DE)

To accurately portray the development status of digital finance and conduct in-depth research in the digital field, we must establish scientific indicators to measure the degree of development of digital finance within a region. It is inaccurate to consider only the breadth of digital account coverage within an area. Still, it is more important to consider the deepening of the use of digital finance in each region to more accurately portray the development status of digital finance. In this paper, we use the depth of use index in the Digital Financial Inclusion Index jointly compiled by the Digital Finance Research Center of Peking University and Ant Financial Services Group to describe the development of digital finance in each region of China. The study contains a comprehensive description of the current development and evolution of digital finance in China and has been used in much literature on digital finance.

The index is based on data from Ant Financial Services’ transaction account data, a comprehensive measure of the current development of digital finance in China at all levels, and has strong credibility. Moreover, its educational value is becoming more and more apparent as the number of people using the data for application research increases. The Digital Inclusive Finance Index contains three major dimensions: breadth of coverage, depth of use, and degree of digitization within this index system. In this paper, we measure the development of digital finance in China by using the depth of use, which is a secondary indicator in the Chinese digital inclusive finance system. The depth of use index covers six business modules, namely, payment, credit, insurance, investment, money fund, and recognition, which summarize all aspects of digital finance in modern economic and social development, and thus we believe that the depth of use index within a region can well represent the development of digital finance in that region.

#### (3) Control variables

The level of economic development (GDP), expressed as GDP per capita. Considering that the existence of an inverted U-shaped relationship between economic growth and income distribution has been verified in previous literature, both the primary term (rgdp) and the second term (rgdpsq) of GDP per capita are included in the model in this paper. Foreign direct investment (fdi) is obtained as the share of the actual foreign direct investment utilized by each prefecture-level city in the regional GDP. The external openness (open) is measured as the share of total import and export trade within prefecture-level cities in the GDP of prefecture-level cities. Urbanization level (urb), measured as the proportion of the urban resident population to the total population at the prefecture level. Internet penetration rate (user) is measured by the ratio of Internet users to a municipality’s total population at the prefecture-level. Financial development level (FD), the article uses the related financial ratio—FIR indicator (deposit and loan balance of financial institutions/GDP of each prefecture) to measure the economic development level of the region. For education level (edu), the article draws on previous literature. It uses the ratio of the number of students enrolled in public secondary and elementary school in prefecture-level municipalities to the total population of the prefecture-level city as a measure of the education level of the region.

### 2. Data sources

Considering the availability and consistency of data, this paper takes 2011–2020 as the sample period. Therefore, it excludes missing samples, covering a total of 280 prefecture-level cities in 28 provinces in mainland China, including the Digital Financial Development Index from the Digital Inclusive Finance Development Report compiled by Peking University, and the data required for other variables from the China City Statistical Yearbook, the provincial and prefecture-level city statistical yearbooks, The other variables are obtained from the China City Statistical Yearbook, local and prefectural-level municipal statistical yearbooks, prefectural-level municipal “Government Work Report” and prefectural-level municipal “National Economic and Social Development Statistical Bulletin.” The regression analysis was conducted using STATA15.1 software, and the descriptive statistics of the main variables of interest are given in Table 1 below.

**Table 1:** Descriptive statistics of the main variables.

Variables	Mean	Standard deviation	Minimum value	Maximum value
*GINI*	0.453	0.068	0.138	0.782
*DE*	153.184	65.148	4.186	325.357
*rgdp*	4.995	3.538	0.568	53.598
*fdi*	0.285	0.378	0	3.859
*open*	0.218	0.637	0	9.259
*urb*	55.956	14.558	0.412	100
*user*	1.063	0.828	0.245	9.659
*FD*	2.549	1.868	0	27.359
*edu*	0.168	0.318	0.058	0.549

### 3. Model construction

This paper first constructs the following linear panel model to verify the linear relationship between the development of digital finance and the distribution of residents’ income.

$$GINI_{it}=\beta_{0}+\beta_{1}*DE_{it}+\beta_{2}CV_{it}+\mu_{i}+\varepsilon_{it}$$ (1)

where: i and t represent each prefecture and year, respectively; *DE*_*it*_ represents the digital financial development level of area i in year t; *CV*_*it*_ represents a set of control variables affecting the income gap in the region; *μ*_*i*_ represents the unobserved factors associated with each prefecture that do not vary over time; *ε*_*it*_ is white noise, meaning the random error term obeying a standard normal distribution.

At different stages of social development, the development of digital finance presents a differentiated impact on the income distribution of the population. At the primary set of digital financial products, not everyone can afford to pay for computers and Internet devices such as cell phones due to the limitations of socio-economic development. The first to use Internet technology and participate in digital finance is a group of wealthy people. The poor cannot access digital financial services because they cannot pay for essential equipment. Therefore, there is a wealth threshold for participation in digital finance. However, the rich do not lack the cost of paying for electronic devices and thus can enjoy digital financial services without a threshold, often obtaining higher returns from digital finance. The continuous development of digital finance further relaxes the credit constraints of the wealthy. As a result, it increases the rate of return that the rich can obtain by renting out their capital, further advancing the wealth accumulation of the wealthy.

In many cases, the poor cannot start and invest freely due to financial constraints, thus limiting their capital gains. In the initial stage of digital finance development, the advancement of Internet technology will increase total social wealth. Still, this benefit is more favorable to the wealthy class, and the increased wealth is less in the pockets of the poor, so the income gap is widened. However, as economic development continues to rise, digital finance development is further improved, the access threshold is gradually lowered, and the poor also have access to the Internet and digital financial services. Thus the credit constraints of the poor are more comprehensive than before, and they can use more funds for entrepreneurship, which slows down the income gap with the wealthy class.

Overall, in the initial development stage of digital finance, the income inequality within the region will deteriorate, and digital finance hurts the regulation of income distribution within the area. In contrast, only when digital finance rises to a particular stage within the region, its subsequent development will help adjust the internal income distribution gap. Based on the above analysis, we introduce the quadratic term of digital finance development and construct the Kuznets model about residents’ digital finance development and income distribution.

Based on the initial regression of the linear model above, the paper proceeds to test the Kuznets effect of the impact of digital financial development on income distribution. The model is set up as follows.


$$\text{GIN}I_{it}=\alpha_{0}+\alpha_{1}*DE_{it}+\alpha_{2}*DE_{it}sq+\alpha_{3}*CV_{it}+\mu_{i}+\varepsilon_{it}$$ (2)

Here, the primary and secondary terms of digital financial development are simultaneously included in the regression equation. If *α*_1_ > 0 and *α*_2_ < 0 hold simultaneously, the bell-shaped relationship between digital economic development and income distribution is verified.

## IV. Results and discussion

### 1. Linear model regression results

In this paper, the regression equations are estimated in the mixed model (Pooled), random effects model (RE), and fixed effects model (FE), and here, to make the coefficients more intuitive, we divide the index of digital financial development level (DE) by 100, adjust it to a benchmark variable of 1, and multiply the Gini coefficient by 100. The regression results are shown in Table 2. Here, the level of digital financial development (DE) is taken as the core explanatory variable, and (1)-(3) are the estimated results obtained from the model after including a series of control variables. The main focus here is on the effect of the core variable, the level of digital financial development (DE).

**Table 2:** Estimation results of the basic model.

	(1)	(2)	(3)
	Pooled	RE	FE
DE (Digital Financial Development Level)	2.273***	2.273***	1.885***
	-16.808	-16.815	-10.945
rgdp (GDP per capita)	0.291***	0.291***	0.291***
	-3.598	-3.605	-3.305
rgdpsq (square of GDP per capita)	-0.00485***	-0.00485***	-0.00463***
	(-2.90)	(-2.90)	(-2.62)
fdi (foreign direct investment)	-0.637**	-0.637**	-0.625**
	(-2.06)	(-2.06)	(-1.94)
open (level of openness to the outside world)	-0.078	-0.085	-0.0908
	(-0 36)	(-0.36)	(-0.32)
urb (level of urbanization)	0.0571***	0.057**	0.154**
	-3.158	-3.165	-5.615
user (Internet penetration rate)	-0.363	-0.37	-0.469
	(-1.17)	(-1.17)	(-1.25)
FD (level of financial development)	0.185-	0.185**	0.223**
	-2.318	-2.325	-2.645
edu (level of education)	-8.931**	-8.932**	-9.975**
	(-2.10)	(-2.10)	(-2.04)
constant	40.52**	40.57**	35.94**
	-41.638	-41.645	-24.155
R^2^ (adjusted)	0.249	0.288	0.27

Note: Data in parentheses are t-statistics,

* p < 0.1,

** p < 0.05,

*** p < 0.01.

For the choice of the panel data model to be estimated, we first calculate the statistics to test whether to choose the fixed effects model or the random-effects model. Hausman’s test value is 26.10, and the corresponding p-value is 0.001, which rejects the original hypothesis. Thus, the fixed effects model is chosen, so column (3) results are more reliable. Therefore, the coefficient estimate of DE here is 1.885, which means that, on average, a one-unit increase in the digital financial development index, controlling for other variables, will increase social income inequality measured by the Gini coefficient by 0.0001885 units. Among the control variables, the coefficients of the rgdp and rgdpsq variables are positive and negative, the urb and FD variables are significantly positive, the fdi and edu variables are very harmful, and the coefficients of the open and user variables are negative but not significant.

According to the estimation results of the basic model, it can be seen that there is a more significant positive relationship between digital financial development and income disparity, and digital economic development is a major influencing factor for the widening of the wealth gap in China in recent years. The results of the empirical analysis are in line with the current domestic concerns about the digital divide arising in the digital economy, where the development of digital finance over the past decade has not narrowed the current income gap in China but instead has worsened the wealth gap in the country. The small amount of literature currently has pointed out the relevant impact of digital financial development on income distribution. Still, little literature has examined it from the perspective of the overall income distribution gap. This paper confirms the close relationship between the two using prefecture-level city data in China, similar to the findings of the currently existing studies on digital finance widening the urban-rural income gap.

### 2. Kuznets model regression results

To investigate whether the effect of digital financial development on income distribution shows a Kuznets effect, we estimate the relationship using a mixed regression model (Pooled), a random-effects model (RE), and a fixed-effects model (FE), respectively, using the same F-test and Hausman test, and conclude that a fixed-effects model should be chosen.

The focus here is on the impact of digital financial development on income disparity. From the regression results in Table 3, the primary term coefficient of DE is significantly positive. The second term coefficient is very harmful, confirming the bell-shaped effect of digital financial development on residents’ income gap. With the continuous development of digital finance, income inequality within regions shows a phenomenon of deterioration before improvement. In addition, the direction of other control variables on income distribution is consistent with the estimation results in the previous chapter. The Kuznets effect exists between economic development and income distribution; foreign direct investment can effectively alleviate the current problem of excessive income disparity; the increasing level of urbanization and financial development will worsen the current status of the income distribution. The reason for the less significant effect of education level on income distribution may be that the proportion of primary and secondary school students in a region is not a good proxy for the education level in the area, and I have not found a more suitable proxy variable due to my limited academic level.

**Table 3:** Estimation results of the nonlinear model.

	(1)	(2)	(3)
	Pooled	RE	FE
DE (Digital Financial Development Level)	3.837***	3.837***	3.279***
	-8.308	-8.315	-6.755
DEsq (Digital financial development level squared)	-0.497**	-0.497**	-0.433***
	(-3.55)	(-3.55)	(-3.06)
rgdp (GDP per capita)	0.323**	0.323**	0.316**
	-3.978	-3.985	-3.585
rgdpsq (square of GDP per capita)	-0.005374**	-0.00537**	-0.00503**
	(-3.21)	(-3.21)	(-2.84)
fdi (foreign direct investment)	-0.674**	-0.674**	-0.662**
	(-218)	(-2.18)	(-2.06)
open (level of foreign opening)	-0.0409	-0.0479	-0.0506
	(-0.23)	(-0.23)	(-0.12)
urb (level of urbanization)	0.0546**	0.0546**	0.146**
	-3.028	-3.035	-5.295
user (Internet penetration rate)	-0.597*	-0.597*	-0.645*
	(-1.79)	(-1.79)	(-1.75)
FD (level of financial development)	0.204**	0.204**	0.234**
	-2.568	-2.575	-2.785
edu (level of education)	-5.007	-5.014	-5.868
	(-1.15)	(-1.15)	(-1.15)
constant	39.25**	39.25**	35.034**
	3.837***	3.837***	3.279***
R^2^ (adjusted)	-8.308	-8.315	-6.755

Note: Data in parentheses are t-statistics,

* p < 0.1,

** p < 0.05,

*** p < 0.01.

In addition, this paper calculates the inflection point of the bell curve, whose value is about DE = 390, which corresponds to the practical implication that the development of digital finance and income inequality are correlated in the prefecture-level cities with the depth of digital finance usage index below 390. In contrast, the growth of digital finance and income inequality are negatively correlated in the prefecture-level towns with the depth of digital finance usage index above 390. The study shows that during the sample period, only Shanghai’s Digital Financial Depth of Use Index exceeds 390. Thus, most regions in China are still on the left side of the bell curve, i.e., the income gap will further widen as the digital economy grows. At present, China is still quite far from crossing the inflection point of the bell curve.

### 3. Robustness test

In the model, the explanatory variables may be correlated with the stochastic disturbance terms, thus creating an endogeneity problem, which may lead to serious bias in the study results, so we need to correct the endogeneity problem. Here we re-estimate the endogenous explanatory variable DE with a one-period lag of LDE as an instrumental variable to replace the current period. Table 4 shows the estimation results of each regression equation. The regression results show that the coefficients of the primary and quadratic terms of the core explanatory variable—the level of digital financial development DE lagged one period are one positive and one negative respectively, reaching the 1% significance level. The signs of the coefficients of the remaining control variables are consistent with the above, indicating that the conclusions obtained from the above model estimation are robust, i.e., the impact of digital financial development on There is a Kuznets effect on the result of digital economic growth on the distribution of residents’ income.

**Table 4:** Robustness test results.

Lagged period of the variable	(1)	(2)	(3)
	Pooled	RE	FE
LDE (level of digital financial development)	4.000***	4.000***	3.559***
	-8.148	-8.155	-6.685
LDEsq (square of the level of digital finance development)	-0.620***	-0.620***	-0.565***
	(-4.12)	(-4.12)	(-3.69)
Constant	41.35***	41.35***	38.13***
	-37.158	-37.165	-22.685
R^2^ (after adjustment)	0.213	0.206	0.18

Note: Data in parentheses are t-statistics,

* p < 0.1,

** p < 0.05,

*** p < 0.01.

### 4. Urban-rural based heterogeneity analysis

In this subsection, we subdivide the Gini coefficients of prefecture-level cities into urban and rural areas to analyze the heterogeneity of different regions. In the past, the attention to income distribution was often focused on the income gap between urban and rural areas but less on the income gap between urban and rural areas. This paper analyzes the correlation between digital finance development and income distribution within urban and rural areas, respectively, with the following equations.


$$GINIci_{it}=\alpha_{0}+\alpha_{1}*DE_{it}+\alpha_{2}*DE_{it}sq+\alpha_{3}*CV_{it}+\mu_{i}+\varepsilon_{it}$$ (3)

$$GINIco_{it}=\alpha_{0}+\alpha_{1}*DE_{it}+\alpha_{2}*DE_{it}sq+\alpha_{3}*CV_{it}+\mu_{i}+\varepsilon_{it}$$ (4)

The results in Table 5 show a Kuznets effect of digital finance on the income gap within urban and rural areas. However, the impact of digital finance development on the income distribution of urban residents is more significant, and the opening of the bell-shaped curve is more minor. This is because the Internet penetration rate of urban groups is higher than that of rural groups. In addition, the depth of Internet use is more significant in urban groups than in rural groups. Therefore, it is easy to obtain that the impact of digital financial development on income distribution in urban areas is more significant than that in rural areas.

**Table 5:** Urban-rural heterogeneity in digital finance and income distribution.

Lagged period of the variable	(1)	(2)
	Urban residents	Rural residents
DE (level of digital financial development)	3.844***	3.096***
	-6.048	-4.855
DEsq (square of the level of digital financial development)	-0.585***	-0.382**
	(-3.18)	(-2.07)
Constant	35.25***	32.63***
	-17.838	-16.475
R^2^ (after adjustment)	0.169	0.187

Note: Data in parentheses are t-statistics,

* p < 0.1,

** p < 0.05,

*** p < 0.01.

Moreover, there are more traditional industries in rural areas, and new Internet-related sectors are concentrated in urban areas, making the impact of digital finance development on the income differentiation of urban residents even greater. Thus, we should pay more attention to the disadvantaged groups in urban areas who do not have access to the Internet and consciously strengthen the Internet education efforts so that more and more people can enjoy the dividends brought by digital finance and eliminate the intra-urban digital divide. Furthermore, for the disadvantaged groups of the Internet, we should provide employment assistance promptly to promote the employment of non-Internet people by the Internet industry, such as online domestic help companies.

## V. Analysis of the threshold effect of digital finance development

### 1. Threshold regression model setting

Following the conceptualization of the (Bruce et al., 1999) threshold model, this paper develops a single panel threshold model of digital financial development and income distribution.

$$GINI_{it}=\beta_{0}+\beta_{1}*DE_{it}I\left(q_{it}\leq \gamma \right)+\beta_{2}*DE_{it}I\left(q_{it}>\gamma \right)+\beta_{3}CV_{it}+\mu_{i}+\varepsilon_{it}$$ (5)

with a dual-panel threshold model:

$$\begin{array}{l} GINI_{it}=\beta_{0}+\beta_{1}*DE_{it}I\left(q_{it}\leq \gamma_{1}\right)+\beta_{2}*DE_{it}I\left(\gamma_{1}<q_{it}\leq \gamma_{2}\right)+\\ \beta_{3}*DE_{it}I\left(q_{it}>\gamma_{2}\right)+\beta_{4}CV_{it}+\mu_{i}+\varepsilon_{it} \end{array}$$ (6)

Where: the subscripts i, t corresponds to the prefecture-level city and the corresponding year, respectively; *GINI* denotes the Gini coefficient; *DE* denotes the development level of regional digital finance; *I*(*q*_*it*_ ≤ *γ*) is the indicative function; *γ* is the threshold value of the single panel threshold model; *γ*_1_, *γ*_2_ is the first threshold value and the second threshold value of the dual panel threshold model, respectively; the remaining control variables are consistent with the previous section of the article.

### 2. Description of threshold variables

Combining the existing literature with the current social development, we set the level of economic growth as a threshold variable to verify the threshold effect of the digital financial product on income distribution in China. The classic literature [52] discusses the differential effects of financial development on income distribution under different economic development conditions, leading to the different effects of digital financial development on income distribution. Digital financial participation requires specific electronic devices and certain Internet knowledge, so digital financial product has a different impact on income distribution in other regions under varying levels of economic development. When the local economy is relatively backward, only a few wealthy people can obtain mobile Internet devices to successfully participate in various activities of digital finance and generate investment income. In contrast, most people are excluded from the threshold of digital finance, and the income gap will be further widened at this time. As society continues to develop and people’s income levels continue to rise, more and more people have access to mobile devices and basic Internet knowledge, digital finance is rapidly expanding among the population, and people are participating in various activities of digital finance, widening their income channels. As a result, the income gap within the region will continue to narrow. Currently, the Internet infrastructure in many poor areas is not well developed, and the lack of Internet base stations hinders residents’ access to the Internet. With the continuous development of the economy, the infrastructure in urban and rural areas becomes more and more complete, and more residents can enjoy the convenience and benefits brought by Internet finance without any obstacles. The poor people can thus achieve higher income and escape from poverty. The above analysis shows that the level of economic development within a region is an essential factor affecting the distribution effect of digital finance.

### 3. Results and discussion

The estimation results of the threshold model are reported in Table 6. The results show a significant nonlinear characteristic of the impact of digital financial development on income distribution. We divide three major income blocks by the threshold variable—GDP per capita corresponding to the calculated threshold value, focusing on the differences in the impact of digital financial development on income distribution under regions with different levels of economic growth. The regression results show that when the per capita income does not exceed the first threshold value of $3172.2, the impact coefficient of digital financial development is 1.743 and is significant at the 1% level; when the per capita income exceeds the first threshold value of $3172.2 but is lower than the second threshold value of $10,344, the estimated coefficient of digital financial development increases from 1.743 to 2.329, indicating that when the regional income level rises to this region, the negative effect of digital economic development on income distribution increases; when the per capita income crosses the second threshold value of $10,344, the coefficient of the digital financial product on income distribution decreases to 2.094, indicating that the negative effect of digital financial development on income distribution will be weakened when the regional economic development level increases to a specific interval. The negative impact of the digital financial product on income distribution in the region is the largest when the level of economic development is in the range of $3172.2 to $10,344. Thus, this paper concludes that the effect of the digital financial product on the income distribution of residents is closely related to the economic development level of its location, and digital financial development at different economic levels has other effects on the income gap within the region, showing a significant threshold feature.

**Table 6:** Threshold panel model regression results.

Variables	Coefficient estimates	Standard deviation	t-value	P-value	95% confidence interval
*DE*_*it*_ ⋅ *I* (*q*_*it*_ ≤ *γ*_1_)	1.743	0.204	8.53	0	[1.342,2.143]
*DE*_*it*_ ⋅ *I* (*γ*_1_ < *q*_*it*_ ≤ *γ*_2_)	2.329	0.196	11.86	0	[1.944,2.714]
*DE*_*it*_ ⋅ *I* (*q*_*it*_ > *γ*_2_)	2.094	0.158	13.26	0	[1.784,2.404]

To sum up, under the conditions of low economic development level, digital financial development will widen the income gap within the region, but the impact effect is weak in this period; as the economic development level further increases, the negative impact of digital finance on income distribution will increase; and when the economic development level rises to a particular stage, the negative effect of digital financial development on income distribution begins to weaken. Thus, as a whole, the impact coefficient of digital finance development in each region is positive, which proves that digital finance development is an important reason why the current domestic income distribution tends to deteriorate, and this is precisely consistent with the research findings in the previous section of this paper.

### 4. Regional division

Due to the unevenness of China’s regional economic development, there are more significant differences in the impact of digital financial development in each region. Therefore, to more intuitively grasp the overall effect of the digital financial product on the level of income distribution in China’s provinces, the threshold variable is used here as a criterion to further divide the sample.

According to the corresponding division of China’s regions in Table 7 below, more than half of China’s prefecture-level cities had real per capita incomes below RMB 3,172.2 in 2011. From 2012 to 2018, most prefecture-level cities had per capita incomes in the range of RMB 3,172.2 to RMB 10,344, i.e., the largest range in which the effect of digital financial development widens the income gap, while those with per capita incomes above RMB 10,344 The majority of prefecture-level cities have always been in the minority. Still, there has been a rising trend in recent years. Therefore, prefectures with different levels of economic development can adopt other policies for digital economy development, and further economic development can effectively curb the negative impact of digital financial development on residents’ income distribution.

**Table 7:** 2011–2018 number of prefecture-level cities by threshold.

Year	*rgdp* ≤ 3172.2	3172.2 < *rgdp* ≤ 10344	*rgdp* > 10344
2011	161	100	6
2012	128	138	7
2013	112	156	10
2014	113	157	27
2015	82	180	17
2016	75	180	19
2017	67	211	20
2018	27	219	46
2019	23	224	48
2020	21	235	46

## VI. Conclusions and recommendations

### 1. Conclusion

This paper combines theoretical analysis and classical literature to conduct an econometric test at the prefecture-level city level. Firstly, we collect the primary data of digital financial development and use the statistical yearbook data of each prefecture-level city to measure the Gini coefficient of each region and conduct descriptive statistics on the current status and development trend of the income distribution, which is used as the primary data work for empirical evidence. In terms of econometric tests, firstly, a linear panel model is used to examine the impact of digital financial development on the income distribution of residents, and the article finds that it has a positive effect on the income distribution gap. Then, according to the classical G-J model theory, the paper further examines the Kuznets effect presented by digital financial development and residents’ income distribution, and finds that there is indeed a bell-shaped curve, and conducts heterogeneity analysis on two major regions, rural and urban, and can see that the effect of digital financial development on the income gap within urban areas is greater than that of the income gap within rural areas, which is consistent with the current reality. Finally, the article also uses the threshold model to test the threshold effect of digital financial development on income distribution in different economic development regions, and it can be seen that its impact on income distribution in areas with varying levels of economic growth is different, and there is indeed a double-threshold effect. The main conclusions of this paper are as follows.

First, with the comprehensive promotion of digitization and the increasing coverage of mobile Internet, China’s digital finance development level has increased significantly. Still, the degree of perfection of the digital finance market does not match the degree of growth of the digital product market. While China’s Internet technology has made world-renowned achievements, the domestic digital finance volume has expanded rapidly, and digitalization has been fully applied at all levels, setting off a wave of reform in various industries. However, there is still an apparent problem of uneven development in the digital finance market, with different classes of people not facing the same digital services. In addition, there are still significant differences in digital finance development between and within regions, with the eastern coastal cities significantly better developed than the central and western regions. Still, the gap is narrowing with the continuous development and improvement of the overall domestic market.

Second, along with the continuous development of digital finance in China, the income distribution of domestic residents tends to deteriorate at this stage. This paper calculates the Gini coefficient of each region to measure the income distribution gap within the area. The Gini coefficient shows an increasing trend, indicating that China’s overall income inequality situation is more severe and the income gap is in a state of further expansion. The Gini coefficient is lower in the backward western regions of China, followed by the eastern provinces and the highest in the central areas.

Third, there is a Kuznets effect on the impact of digital financial development on the income distribution of residents in China. The difference influences the impact of digital financial development on income distribution in the level of economic development within regions with significant threshold characteristics. There is a bell-shaped curve relationship between the degree of digital financial development measured by the depth of digital financial use and the Gini coefficient of prefecture-level cities, indicating that as the level of digital economic growth continues to increase, the income gap within regions in China shows a trend of change that first increases and then decreases. On the whole, the product of digital finance in China is not yet perfect, and most of the prefecture-level cities have not yet crossed the inflection point of the bell curve but are still in the upward range on the left side of the curve. Nevertheless, the impact of digital finance development on income disparity shows a significant positive correlation. The level of income inequality will further increase with the development of regional digital finance. In addition, the effect of the digital financial product on income distribution is influenced by the level of economic development within the region. Regions at different stages of economic growth have differential effects of their digital financial development on income distribution within the area and show non-monotonic bithreshold characteristics: its adverse effects first gradually increase with the rise of economic development level, and they tend to weaken. Thus, the impact of digital financial development on income distribution in each region of China is not an isolated process but works together and interacts with local socio-economic development.

### 2. Recommendations

First, to narrow the divide created by different regions in digital development from the source. Due to the unbalanced nature of China’s economic growth, the social population has gradually opened up gaps in income and education, resulting in the differentiation of various classes. The inequality in income and education will lead to cracks in access to information and data processing among different groups in different regions in the current wave of informationization, thus causing the digital divide phenomenon. To solve the current digital range faced, the focus is to vigorously promote social equity, focus on solving the problem of uneven development among various regions and groups, and pay more attention to the education problems in the poor areas of central and western China and the rural areas, actively carry out policy promotion, attract educational resources to enter, improve the level of the human capital of groups in less developed regions, and enhance the ability of residents in the area to have equal access to information technology.

Second, accelerate the digitization process and coordinate policies related to opening up to the outside world, urbanization, Internet penetration, and financial development so that policies are biased toward less developed regions and increase their information dividends. Each part should speed up the local information construction and give better play to the advantages of Internet technology so that the development of local digital finance can achieve the inflection point crossing as early as possible and reduce the income gap within the region.

Thirdly, we should take advantage of the matching and complementary relationship between digital financial development and other economic systems to effectively play the positive role of digital technology in improving income distribution. As mentioned in the text, the impact of digital financial development on income distribution within a region is not isolated, and the human capital and physical capital that individuals have will also interact with digital financial development. Therefore, when formulating policies on digital finance development, government departments should not only regard digital finance as an independent variable affecting regional income distribution but also ignore the correlation with other development factors. The main aspects are as follows: (1) to improve the level of local economic development and further increase the income level of residents; (2) to promote the regional balance of financial expenditure on education, to promote the development of education in a balanced direction and to realize the equity of educational opportunities at all levels; (3) to continue to attract foreign investment and optimize the trade structure, to promote the flow of financial expenditure to less developed regions and disadvantaged groups. All of the above measures can promote the development of China’s income distribution in the direction of equity.

## Supporting information

S1 Data (XLSX)

## References

[1] Towse R. Economics of music publishing: copyright and the market. J Cult Econ. 2017;41: 403–420.

[2] Li J, Wu Y, Xiao JJ. The impact of digital finance on household consumption: Evidence from China. Econ Model. 2020;86: 317–326.

[3] Tse-Chun L, Lu X. How Do Short-Sale Costs Affect Put Options Trading? Evidence from Separating Hedging and Speculative Shorting Demands. Rev Financ. 2016; 5.

[4] Gould DM, Melecky M, Panterov G. Finance, growth and shared prosperity: Beyond credit deepening. J Policy Model. 2016;38: 737–758.

[5] Ono T. Public education and social security: a political economy approach. Econ Gov. 2015;16: 1–25.

[6] Munyegera GK, Matsumoto T. Mobile Money, Remittances, and Household Welfare: Panel Evidence from Rural Uganda. World Dev. 2016;79: 127–137.

[7] Ying GE, Xue J. THE IMPACT OF THE ANTI-CORRUPTION CAMPAIGN ON INCOME DISTRIBUTION: EVIDENCE FROM CHINA. Singapore Econ Rev. 2021.

[8] Sathar F, Dalvie M, Rother HA. Review of the Literature on Determinants of Chemical Hazard Information Recall among Workers and Consumers. Int J Environ Res Public Health. 2016;13. doi: 10.3390/ijerph13060546 27258291 PMC4924003

[9] Wang Z, Zhang D, Wang J. How does digital finance impact the leverage of Chinese households? Appl Econ Lett. 2021; 1–4.

[10] Wang X, He G. Digital Financial Inclusion and Farmers’ Vulnerability to Poverty: Evidence from Rural China. Sustainability. 2020;12: 1668.

[11] Malik Samreen. Financial-integration thresholds for consumption risk-sharing. Int Rev Econ Financ. 2015.

[12] Allegret JP, Azzabi S. Finance, growth and threshold effects. Panoeconomicus. 2012;59: 553–581.

[13] Fernández-Olit B, Paredes-Gázquez JD, Cuesta-González MDL. Are Social and Financial Exclusion Two Sides of the Same Coin? An Analysis of the Financial Integration of Vulnerable People. Soc Indic Res. 2018;135: 1–24.

[14] Martin-Oliver A. Financial exclusion and branch closures in Spain after the Great Recession. Reg Stud. 2019;53: 562–573.

[15] Tam T, Jiang J. Divergent Urban-Rural Trends in College Attendance: State Policy Bias and Structural Exclusion in China. Sociol Educ. 2015;88: 160–180.

[16] Balcázar Felipe C. Lower bounds on inequality of opportunity and measurement error. Econ Lett. 2015;137: 102–105.

[17] Foguel MN, Veloso FA. Inequality of opportunity in daycare and preschool services in Brazil. J Econ Inequal. 2014;12: 191–220.

[18] Korupp SE, Marc S. Causes and Trends of the Digital Divide. Eur Sociol Rev. 2005; 409–422.

[19] Martin SP, Robinson JP. The Income Digital Divide: Trends and Predictions for Levels of Internet Use. Soc Probl. 2007;54: 1–22.

[20] H TN, R AD, D K, W ML. An examination of seven years of technology integration in Florida schools: Through the lens of the Levels of Digital Divide in Schools. Comput Educ. 2017;113: 135–161.

[21] Ho SY, Iyke BN. Determinants of stock market development: a review of the literature. Stud Econ Financ. 2017;34: 143–164.

[22] Sun J, Luo H. Evaluation on equality and efficiency of health resources allocation and health services utilization in China. Int J Equity Health. 2017;16: 127. doi: 10.1186/s12939-017-0614-y 28709422 PMC5513103

[23] Lin Y, Zhang Q, Chen W, Ling L. The social income inequality, social integration and health status of internal migrants in China. Int J Equity Health. 2017;16: 1–11. 28778201 10.1186/s12939-017-0640-9 PMC5545016

[24] Ren B, Li L, Zhao H, Zhou Y. THE FINANCIAL EXCLUSION IN THE DEVELOPMENT OF DIGITAL FINANCE—A STUDY BASED ON SURVEY DATA IN THE JINGJINJI RURAL AREA. Singapore Econ Rev. 2018;63: 1850001.

[25] Alhassan A, Li L, Reddy K, Duppati G. The impact of formal financial inclusion on informal financial intermediation and cash preference: evidence from Africa. Appl Econ. 2019;51: 4597–4614.

[26] Hong M, Zhang W. Industrial structure upgrading, urbanization and urban-rural income disparity: evidence from China. Appl Econ Lett. 2020; 1–6.

[27] Su CW, Liu TY, Chang HL, Jiang XZ. Is urbanization narrowing the urban-rural income gap? A cross-regional study of China. Habitat Int. 2015;48: 79–86.

[28] Callander EJ, Schofield DJ, Shrestha RN. Capacity for Freedom—A New Way of Measuring Poverty Amongst Australian Children. Child Indic Res. 2012;5: 179–198.

[29] Clark C, Gorski P. Multicultural Education and the Digital Divide: Focus on Socioeconomic Class Background. Multicult Perspect. 2002;4: 25–36.

[30] John H, Huang J, James K, Bruce F, Robert M, Joseph S, et al. Use of e-Health Services between 1999 and 2002: A Growing Digital Divide. J Am Med Informatics Assoc. 2005; 164–171. 10.1197/jamia.M1672 PMC551548 15561786

[31] Aminuzzaman S, Baldersheim H, Jamil I. Talking back! Empowerment and mobile phones in rural Bangladesh: a study of the village phone scheme of Grameen Bank. Contemp South Asia. 2003;12: 327–348. doi: 10.1080/0958493032000175879

[32] Donner R, Seehafer N, Sanjuán MAF, Feudel F. Low-dimensional dynamo modelling and symmetry-breaking bifurcations. Phys D Nonlinear Phenom. 2006;223: 151–162.

[33] Devins D, Darlow A, Smith V. Lifelong Learning and Digital Exclusion: Lessons from the Evaluation of an ICT Learning Centre and an Emerging Research Agenda. Reg Stud. 2002;36: 941–945.

[34] Baker FC, Colrain IM, Trinder J. Reduced parasympathetic activity during sleep in the symptomatic phase of severe premenstrual syndrome. J Psychosom Res. 2008;65: 13–22. doi: 10.1016/j.jpsychores.2008.04.008 18582607 PMC2519123

[35] Jiang Y, Shi X, Zhang S, Ji J. The threshold effect of high-level human capital investment on China’s urban-rural income gap. China Agric Econ Rev. 2011;3: 297–320.

[36] Madyun N, Lee MS. The Influence of Female-Headed Households on Black Achievement. Urban Educ. 2010;45: 424–447.

[37] Luo X, Caron J, Karplus VJ, Zhang D, Zhang X. Interprovincial Migration and the Stringency of Energy Policy in China. Energy Econ. 2016;58: 164–173.

[38] Aricat RG. Is (the study of) mobile phones old wine in a new bottle? A polemic on communication-based acculturation research. Inf Technol People. 2015;28: 806–824.

[39] Beveridge C, Joshi M. THE EFFICIENT COMPUTATION OF PRICES AND GREEKS FOR CALLABLE RANGE ACCRUALS USING THE DISPLACED-DIFFUSION LMM. Int J Theor Appl Financ. 2014;17: 1–218.

[40] Jensen R. The Digital Provide: Information (Technology), Market Performance, and Welfare in the South Indian Fisheries Sector. Q J Econ. 2007;122: 879–924.

[41] Muto M, Yamano T. The Impact of Mobile Phone Coverage Expansion on Market Participation: Panel Data Evidence from Uganda. World Dev. 2009;37: 1887–1896.

[42] Islam MA, Mostak K, Hoq G. Community Internet Access in Rural Areas: A study on Community Information Centres in Bangladesh. Malaysian J Libr Inf Sci. 2010;15: 109–124.

[43] Park CY, Mercado RJR. FINANCIAL INCLUSION, POVERTY, AND INCOME INEQUALITY. Singapore Econ Rev. 2018;Forthcomin: 419–435.

[44] Li Z, Griffin-Shirley N, Kelley P, Banda DR, Smith DW. The Relationship Between Computer and Internet Use and Performance on Standardized Tests by Secondary School Students with Visual Impairments. J Vis Impair Blind. 2012;106: 609–621.

[45] Goldberg WA, Lucas-Thompson RG. College Women Miss the Mark When Estimating the Impact of Full-Time Maternal Employment on Children’s Achievement and Behavior. Psychol Women Q. 2014;38: 490–502.

[46] Salia Mahamadu, Nsowah-Nuamah Nicholas N. N, William, et al. Effects of Mobile Phone Use on Artisanal Fishing Market Efficiency and Livelihoods in Ghana. Electron J Inf Syst Dev Ctries. 2011;47: 1–20.

[47] Atasoy H. The Effects of Broadband Internet Expansion on Labor Market Outcomes. Ind Labor Relat Rev. 2013;66: 315–345.

[48] Acemoglu D, Restrepo P. Secular Stagnation? The Effect of Aging on Economic Growth in the Age of Automation. Am Econ Rev. 2017;107: 174–179. doi: 10.1257/aer.p20171101

[49] Brynjolfsson E, Mitchell T. What can machine learning do? Workforce implications. Science (80-). 2017;358: 1530–1534. doi: 10.1126/science.aap8062 29269459

[50] Yan Y, Ping G. Farmers’ financial choices and informal credit markets in China. China Agric Econ Rev. 2012;4.

[51] C TL, L L, O S. Does banking competition alleviate or worsen credit constraints faced by small- and medium-sized enterprises? Evidence from China. J Bank Financ. 2013;37: 3412–3424.

[52] Bauer BO, Greenwood B. Modification of a linear bar-trough system by a standing edge wave. Mar Geol. 1990;92: 177–204.

